# Maize plants can enter a standby mode to cope with chilling stress

**DOI:** 10.1186/s12870-016-0909-y

**Published:** 2016-10-04

**Authors:** Laëtitia Riva-Roveda, Brigitte Escale, Catherine Giauffret, Claire Périlleux

**Affiliations:** 1 Arvalis – Institut du Végétal, Service Génétique, Physiologie et Protection des Plantes, Chemin de Pau 21, F-64121 Montardon, France; 2 UMR SADV, INRA, Université de Lille 1 Sciences et Technologies, F-80203 Estrées-Mons, France; 3 UR AgroImpact, INRA, F-80203 Estrées-Mons, France; 4 InBioS, PhytoSYSTEMS, Laboratory of Plant Physiology, University of Liège, Sart Tilman Campus Quartier Vallée 1, Chemin de la Vallée 4, B-4000 Liège, Belgium

**Keywords:** Maize (*Zea mays*), Cold tolerance, Leaf growth, Photoprotection

## Abstract

**Background:**

European Flint maize inbred lines are used as a source of adaptation to cold in most breeding programs in Northern Europe. A deep understanding of their adaptation strategy could thus provide valuable clues for further improvement, which is required in the current context of climate change. We therefore compared six inbreds and two derived Flint x Dent hybrids for their response to one-week at low temperature (10 °C day/7 or 4 °C night) during steady-state vegetative growth.

**Results:**

Leaf growth was arrested during chilling treatment but recovered fast upon return to warm temperature, so that no negative effect on shoot biomass was measured. Gene expression analyses of the emerging leaf in the hybrids suggest that plants maintained a ‘ready-to-grow’ state during chilling since cell cycle genes were not differentially expressed in the division zone and genes coding for expansins were on the opposite up-regulated in the elongation zone. In photosynthetic tissues, a strong reduction in PSII efficiency was measured. Chilling repressed chlorophyll biosynthesis; we detected accumulation of the precursor geranylgeranyl chlorophyll *a* and down-regulation of *GERANYLGERANYL REDUCTASE* (*GGR*) in mature leaf tissues. Excess light energy was mostly dissipated through fluorescence and constitutive thermal dissipation processes, rather than by light-regulated thermal dissipation. Consistently, only weak clues of xanthophyll cycle activation were found. CO_2_ assimilation was reduced by chilling, as well as the expression levels of genes encoding phosphoenolpyruvate carboxylase (PEPC), pyruvate orthophosphate dikinase (PPDK), and the small subunit of Rubisco. Accumulation of sugars was correlated with a strong decrease of the specific leaf area (SLA).

**Conclusions:**

Altogether, our study reveals good tolerance of the photosynthetic machinery of Northern European maize to chilling and suggests that growth arrest might be their strategy for fast recovery after a mild stress.

**Electronic supplementary material:**

The online version of this article (doi:10.1186/s12870-016-0909-y) contains supplementary material, which is available to authorized users.

## Background

Tolerance to cold has been a long lasting issue for maize cultivation. Extension from its native tropical area in Southwestern Mexico toward Northern countries indeed required selection of short-cycle varieties to alleviate the prolongation of growth duration by low temperature. Prominent in maize history is the early flowering Northern Flint race that adapted to cold temperate regions of Northeastern America and was introduced in Northern Europe probably at the beginning of the 16th century [1]. Interestingly, in both American and European continents, Northern races were hybridized with late materials to produce new types adapted to mid-latitude climates, such as Corn Belt Dent in America resulting from the inter-crossing between Northern Flint and Southern Dent races [2]. After World War II, traditional landraces were progressively replaced by hybrid varieties [3]. European Flint inbred lines provided valuable traits for regions with cool and wet spring conditions: cold tolerance, early vigor and short growing cycles [4]. Thanks to their good heterotic pattern with American Dent material, they have been widely used in Northern Europe for hybrid production [5].

New challenges give to cold tolerance of maize a renewed interest. The current climate change encourages early planting that potentially increases yield and participates to water deficit avoidance in summer. Early harvesting is also suitable to prevent fungal growth and mycotoxin production in grain and to reduce drying costs. In France, for example, the mean sowing date has advanced from 5 to 15 days over the past 30 years and this trend will probably increase in the future [6]. However, earlier sowing dates increase the risk of exposure of the plants to cold and hence require to re-evaluate cultivated materials and to select inbreds that are more tolerant to low temperatures.

Assessing cold tolerance in maize requires the choice of an experimental design. The conditions of stress occurrence, its intensity and duration as well as the developmental stage of the plants, are all critical parameters that delimit the scope of the research. On the one hand, laboratory experiments were, and still are, instrumental in identifying the physiological and cellular effects of cold. They are generally performed on young seedlings of reference genotypes, often inbred lines such as B73, transferred into cold rooms (<5 °C) for a short period. This kind of approach clearly demonstrated that cold impairs photosynthetic machinery and unveiled the physiological mechanisms of cold tolerance [7, 8]. On the other hand, field experiments explore genetic diversity within large sets of genotypes to reliably estimate the relationship between cold tolerance traits and agronomic performance. Growth of maize is strongly limited below 15 °C [9] and ‘chilling’ commonly refers to the temperature range between 5 and 15 °C [8]. Reduced leaf growth at these temperatures implies that light interception area is limited and, in addition to impaired photosynthesis, contributes to depressed plant productivity in terms of biomass or grain yield [10]. Numerous quantitative traits (QTL) have been identified but were only partially consistent across the different mapping populations used, indicating a strong influence of the genetic background [11]. Moreover, assessment of chilling tolerance in the field is hampered by the fact that fluctuating environments and occurrence of multiple stresses complicate the identification of causal relationships between chilling and final yield. Several authors have reported poor correlations between widely used traits, e.g., vigor of seedlings is neither positively associated with grain yield [12] nor with dry matter accumulation [13]. Selectable phenotypic traits thus remain an issue for breeding cold tolerance in maize.

European Flint inbred lines are still used as a source of adaptation to cold in most maize breeding programs in Northern Europe [3] and hence a deep understanding of their adaptation strategy could provide valuable clues for further improvement. The effects of cold on cellular processes governing leaf growth and photosynthesis can be assessed simultaneously along the maize leaf which shows a longitudinal gradient with proliferative, expanding and mature cells located at increasing distance from the base. New markers could be discovered thanks to the availability of transcriptome profiles associated with this developmental gradient [14] and from homology to known regulatory networks disclosed in Arabidopsis, e.g., cell cycle genes [15] or cold-signalling components [7, 16]. The best understood cold response pathway in plants involves CBF (C-repeat/drought-responsive element binding) transcription factors, also known as DREB1, which activate target genes [17]. ICE1 (inducer of CBF/DREB expression 1) is an upstream regulator of the expression of the *CBF*/*DREB1* genes and is itself activated by cold at the post-translational level [18].

The growth gradient of the maize leaf is established early after initiation and persists after the proliferative zone splits to occupy a region at the base of the blade and at the base of the sheath, separated by the ligule [19]. Chilling can then restrict growth of the blade and/or the sheath of the leaves, by impairing cell proliferation and/or cell expansion, depending on their developmental stage [15, 20]. In addition, when chilling occurs soon after leaf initiation, the reduction of cell division rate can be partly compensated by an increase in cell length [21]. Cold compromises the assembly of the photosynthetic apparatus in differentiating cells [22], or its efficiency and integrity in mature tissues [23, 24]. Cold reduces the velocity of enzymatic reactions such as those catalysed, in C4 species like maize, by phosphoenolpyruvate carboxylase (PEPC), pyruvate orthophosphate dikinase (PPDK), or Rubisco [25]. The resulting decrease in CO_2_ assimilation leads to saturation of the electron transport [26]. Light energy that is absorbed in excess can lead to photooxidation and damage to membrane proteins unless it is re-emitted as chlorophyll fluorescence or dissipated as heat. Changes in fluorescence yield thus reflect changes in photochemical (PSII efficiency, ΦPSII) and non-photochemical quenching (NPQ) of excitation energy and are widely used as physiological proxy of cold tolerance [26]. Heat dissipation is stimulated by the acidification of the thylakoid lumen, which activates psbS, a PSII protein embedded in the thylakoid membrane, and the xanthophyll cycle, i.e., the reversible de-epoxidation of violaxanthin into dissipative zeaxanthin. In case these photoprotective mechanisms are not sufficient, PSII reaction centers are subject to permanent damage, a process called photoinhibition (reviewed in [27]).

The aim of this study was to characterize maize inbreds adapted to temperate climate for their tolerance to chilling. Since prediction of hybrid tolerance from parental inbreds may not be reliable [28], we also analysed derived Flint x Dent hybrids. As we expected subtle differences for cold tolerance in this material, the experiments were performed in controlled growth chambers, which provide an appropriate scale for dissecting the mechanisms of chilling tolerance in reproducible conditions [29]. Since chilling at early stages was often studied in the literature and appeared to have few consequences on later development [13, 21], we focused on the steady-state vegetative growth of the plants. We used an array of physiological, biochemical and molecular parameters in order to gain an overview of the adaptive mechanisms that could account for cold tolerance and help to define selectable phenotypic traits.

## Methods

### Plant material and culture conditions

Three unrelated Flint lines (F2, F283, F03802) released in 1958, 1985 and 2008, respectively, and three Dent lines (F353, B73, Mo17) representing Iodent/European Dent, Stiff Stalk and Lancaster heterotic groups, respectively, were provided by INRA. Two hybrids (F03802xF353, F2xF353) were produced during the growing season 2011–2012 in Graneros (Chile) and during summer 2013 in Montardon (France).

Grains were sown in compressed peat pots (Jiffy pots, Ets Lejeune, Warsage, Belgium) filled with horticultural compost (TYP*ical* Tonerde I, Brill substrate GmbH & Co., Georgsdorf, Germany; www.brill-substrate.com). When the third leaf emerged, seedlings were transplanted into larger pots (14-cm diameter, 10-cm height) filled with the same compost supplemented with 10 % perlite (v/v). A nylon mesh was inserted in the bottom of the pot and was soaked in a reservoir to provide complete nutrient solution continuously (12.6 mEquiv. N l^−1^). Plants were grown in controlled cabinets (Conviron, Winnipeg, Canada) in 16-h long days, 300 μmol m^−2^ s^−1^ (inbred lines) or 400 μmol m^−2^ s^−1^ (hybrids) of photosynthetically active radiation (PAR) being provided at the canopy level by 54-W fluorescent tubes and 40-W incandescent bulbs (ratio 3:1). Air relative humidity was 70 %.

### Temperature treatments for inbreds and hybrids

Standard temperature was 24 °C day/18 °C night. For cold treatment, plants were transferred to 10 °C day/7 °C night (inbred lines) or 10 °C day/4 °C night (hybrids) for one week.

Experimental series comprised 15 plants of each genotype, in control and treated (cold) conditions. The numbers of plants and replications used for all traits measured are detailed in the table and figure legends.

### Growth measurements

The visible leaf stage (VL) was defined as the total number of visible leaves counted with the plant held at eye level. For the youngest emerging leaf, the decimal stage was evaluated as in [30]: the ratio (V/T) between the length of the visible portion (V) and the total length of the leaf measured from its tip to the base of the plant (T) was divided by its maximum value (V/T)_max_ reached at the time the next leaf emerged. In practice, (V/T)_max_ was between 0.47 and 0.60 for the genotypes used in this study. The phyllochron is the time (days) between successive leaf appearance and was calculated as the inverse of mean VL increase rate.

Leaf growth was estimated by leaf elongation rate and final leaf length. Leaf elongation rate was estimated by measuring the elongation of the visible part of the leaf until it reached its final length. In order to avoid cumulating leaf and stem growth in leaf elongation rate calculation, length was measured from the tip of the growing leaf to the ligule of the youngest fully expanded leaf below it. End-point measurement of leaf length was made at 12-VL stage. Specific Leaf Area (SLA, cm^2^ g dry weight^−1^) determination was made on 8-mm diameter leaf discs harvested at mid-length of leaf 4, outside the mid-vein, at the end of the cold treatment.

### Photosynthetic parameters measurements

Photosynthetic performance was evaluated by measuring the maximum quantum yield of photosystem II (Fv/Fm) and quantum efficiencies of photochemistry (ФPSII = 1 – Fs/Fm’), of regulated thermal energy dissipation (ФNPQ = Fs/Fm’ – Fs/Fm) and of constitutive thermal and radiative (fluorescence) energy dissipation (Φf,D = Fs/Fm) [31]. The sum of the three yields (ΦPSII, ΦNPQ and Φf,D) is equal to 1 and reflects light energy partitioning. In practice, we measured fluorescence at mid-length of leaf 4 emerged part after adaptation in darkness (at least 20 min) or in the light (at least 6 min at 300 or 400 μmol m^−2^ s^−1^ PAR) using a Handy PEA fluorimeter (Hansatech). The measurements were performed before (day 0), during (days 1, 3 and 7) and after (day 14) the chilling period.

The net CO_2_ assimilation rate was measured using a LI-6400 infrared gas exchange analyser (LI-COR Inc. Lincoln, NE, USA). CO_2_ assimilation was measured at 400 μmol m^−2^ s^−1^ constant light and 380 ppm constant external CO_2_ concentration, around the midpoint of leaf 4 blade. Measurements were performed at least 4 h after light-on.

### Pigments and sugars analyses

8-mm leaf discs were harvested from leaf 4 blade, 15 cm from the ligule, on either side of the midrib. Sampling was performed 5–7 h after light-on (between 2:00 and 4:00 p.m.).

For pigment extraction, one leaf disc was ground in liquid nitrogen and the powder was extracted twice with 1 ml of methanol (once overnight at −80 °C, once for 30 min at −20 °C). Pooled supernatants were filtered on 0.45 μm syringe filters. 100 μl were used for high-performance liquid chromatography using a Shimadzu set-up (Prominence series, Shimadzu, Kyoto, Japan) comprising a pump (LC-20AT), an autosampler (SIL-20 AC) and a photodiode array detector (SPD-M20A). A Nova Pak C18 column (3.9 × 150 mm, 4 μm pore size) from Waters (Ireland) was used for separation. Acquisition and data analysis were performed using the Empower software (Shimadzu). Separation was obtained with the following program: 2-min gradient from 100 % solvent A (80 % methanol: 20 % 100 mM ammonium acetate pH7) to 100 % solvent B (90 % acetonitrile in water); 23-min gradient from 100 % B to 31 % B : 69 % C (100 % ethylacetate); 10-min gradient from the latter solvent mixture to 100 % A. The solvent flow rate was 1 ml min^−1^. All solvents were HPLC grade and obtained from VWR (Leuven, Belgium); commercial pigments standards from DHI-Water and Environment (Horstholm, Denmark) were used for calibration. Determination of geranylgeranyl-chlorophyll *a* peak was made according to [32]. Total carotenoids include neoxanthin, lutein, β caroten and the 3 pigments of the xanthophyll cycle: violaxanthin (V), antheraxanthin (A) and zeaxanthin (Z). A de-epoxidation index, representing the amount of zeaxanthin formed by conversion of violaxanthin via the intermediate antheraxanthin, was calculated as (A + Z)/(V + A + Z).

For sugar extraction, one leaf disc was ground in liquid nitrogen and the powder was suspended in 1 ml of 80 % ethanol: 20 % 100 mM Hepes-KOH (pH 7.1), 10 mM MgCl_2_ as in [33]. After incubation at 80 °C for 45 min, tubes were cooled down to room temperature and centrifuged 10 min at 4 °C, 13000 g. Pellet and supernatant were used for quantification of starch and soluble sugars, respectively. For starch analysis, the pellet was washed twice with 40 mM sodium acetate pH 4.5, then resuspended in 200 μl of sterile water and autoclaved 2 times for 20 min at 120 °C to solubilize starch. After homogenization, quantification of starch was performed with an enzymatic kit from R-Biopharm/Roche (cat nr 10207748035) following supplier instructions. For soluble sugar analyses, the supernatant was evaporated in speedvac and the dry residue was dissolved in 1 ml of sterile water. Quantification of glucose, fructose and sucrose was performed with an enzymatic kit from R-Biopharm/Roche (cat nr 10716260035). Absorbance was determined at 340 nm with a Lambda 20 UV/VIS spectrophotometer (Perkin Elmer, Norwalk, CT).

### Transcriptional analyses

Samples were collected from leaf 4 mature zone (8-mm discs taken 15 cm from the ligule), leaf 5 division zone (segment 0–1.5 cm from the base) and leaf 5 elongation zone (segment 3–4 cm from the base). Leaf tissues were harvested 5–7 h after light-on (between 2:00 and 4:00 p.m.) and immediately frozen in liquid nitrogen. Samples from 10–15 plants were pooled, ground in liquid nitrogen and stored at −80 °C until use. Total RNA was extracted from 100 mg of leaf tissue with 1 ml TriReagent (Ambion, Applied Biosystems). Two phenol/chloroform extractions were performed with two successive purifications with 3 M potassium acetate pH 5.2 and 3 M sodium acetate pH 5.2. RNA was precipitated with isopropanol overnight at −20 °C. RNA quantity and quality were controlled by absorption measurements at 230 and 260 nm (BioSpec-Nano, Shimadzu). After DNase treatment of total RNA (1 U DNase μg-1) for one hour at 37 °C, first-strand cDNA was synthesized from 1 μg RNA, using MMLV reverse transcriptase and oligo(dT)15 according to the manufacturer’s instructions (Promega). Aliquots were used as templates for qPCR with gene-specific primers (Additional file 1: Table S1). In each 20-μl reaction, 3 μl cDNA and 10 μM of primers were used. Reactions were performed in triplicate using SYBR Green I (Eurogentec) in 96-well plates with an iCycler IQ5 (Bio-Rad). For normalization, a geNormPLUS analysis was previously performed with ten housekeeping genes chosen from the literature [34, 35]. Five genes were selected as constitutive genes (geNorm M value <0.5, see Additional file 2: Figure S1): *CULLIN* (*CUL*), *UBIQUITIN* (*UBI*), *UBIQUITIN CONJUGATING ENZYME* (*UCE*), *FOLYLPOLYGLUTAMATE SYNTHASE* (*FGP1*) and *LEUNIG* (*LUG*). The amplification efficiencies of all primer pairs were between 80 and 117 % (see Additional file 1: Table S1). The Relative Expression Software Tool (REST, version 2009; http://rest.gene-quantification.info/), which operates on Cq values, includes different PCR efficiencies and uses multireference genes for normalization [36, 37], was used for the relative quantification of qPCR data.

### Statistical analyses

Data were analysed with R software (http://www.R-project.org/). We performed Student’s T tests for results obtained from one single experiment; for replicate experiments, we pooled results when possible and performed analysis of variance in combination with means comparison (Tukey method) to distinguish statistically different groups (*P* < 0.05). We fitted our data with linear mixed-effects models, using the lme4 package (http://CRAN.R-project.org/package=lme4), to take into account the effect of independent experiments (random effect).

## Results

### Leaf growth is suspended in cold

**Fig. 1:**
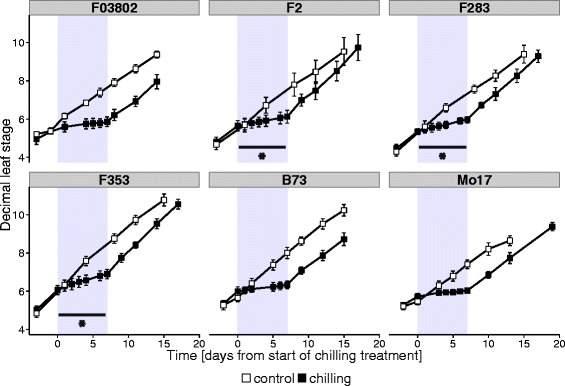
Effect of chilling on leaf appearance rate in 6 inbred lines of maize. The 7-day chilling treatment (10 °C day/7 °C night) was applied at the 5-VL stage (grey zone). Decimal leaf stage was measured on 15 plants per genotype; results shown are means ± sd. Open symbols show plants grown in control conditions; filled symbols show chilled plants. Upper line: Flint inbreds; lower line: Dent inbreds. * indicates significant growth during chilling (slope different from 0)

The growth of 6 inbreds cultivated in phytotronic cabinets at 24 °C day/18 °C night was recorded by regular measurement of the decimal visible leaf (VL) stage (Fig. 1). At the 5-VL stage, one half of the plants were exposed to a chilling-treatment of one week at 10 °C day/7 °C night. We extracted from Fig. 1 the mean phyllochron (time between successive leaf appearance) of inbreds in control conditions, during (between day 0 and 7) and after (from day 7 to 10-VL stage) chilling (Table 1). During chilling, growth was almost arrested. Among the 6 inbreds, the Flint line F03802, as well as the 2 Dent lines B73 and Mo17 showed a complete stop, since the VL stage after the low temperature treatment was not different than before. By contrast, the 3 inbreds F2, F283, and F353 kept on growing very slowly at 10 °C/7 °C. After return to warm conditions, growth recovered in all genotypes up to control rate level (Table 1), so that the impact of the treatment finally appeared as a short delay in VL. An end-point measurement of the final length of leaf 5 was performed at the 12-VL stage: in all genotypes, leaf 5 blade was 55-cm to 70-cm long in control conditions and was 10 % to 18 % shorter in plants that had been chilled for one week (Table 1). This negative effect of cold on leaf length was not visible on shoot biomass measured à 12-VL stage (Table 1).

**Table 1:** Effect of chilling treatment on leaf appearance rate, final length of leaf 5 blade and shoot biomass in 6 inbred lines of maize

Inbred	Phyllochron [days]			Final length of 5th leaf blade [cm]		Shoot dry weight [g]	
	Control conditions	During chilling	After chilling	Control	Chilling	Control	Chilling
F03802	4.0	24	3.3	69.1 ± 2.2	61.9 ± 3.1*	14.5 ± 1.9	16.4 ± 3.9
F2	3.7	15	2.8	66.2 ± 3.9	54.6 ± 2.3*	14.8 ± 2.3	11.8 ± 2.8
F283	3.6	12	3.1	60.3 ± 3.1	51.2 ± 2.4*	16.5 ± 3.4	15.6 ± 3.1
F353	3.0	8.8	2.8	59.3 ± 3.0	50.3 ± 4.2*	13.7 ± 1.4	12.9 ± 1.8
B73	3.6	21	3.3	56.7 ± 2.5	48.0 ± 2.2*	14.1 ± 2.4	12.4 ± 2.0
Mo17	4.3	23	3.6	68.9 ± 3.2	56.2 ± 3.5*	19.9 ± 2.6	18.4 ± 3.1

The 7-day chilling treatment (10 °C day/7 °C night) was applied at 5-VL stage. Measurements of final leaf length and biomass were performed at 12-VL stage. Data are means ± sd of 15 plants. *indicates significant effect of chilling (*P* < 0.05)

**Fig. 2:**
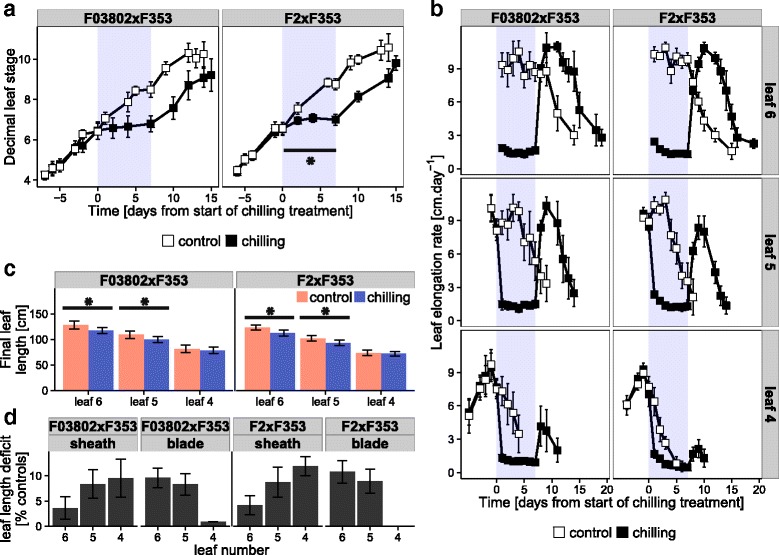
Effect of chilling on leaf growth in two maize hybrids. The 7-day chilling treatment (10 °C day/4 °C night) was applied at about the 6-VL stage (grey zone in a and b). **a** Leaf appearance rate. Decimal leaf stage was measured on 15–20 plants per genotype in each treatment; results shown are means ± sd for 7 experiments. * indicates significant growth during chilling period (slope different from 0). **b** Leaf elongation rate of leaves 4, 5 and 6. Data are means ± sd of 15 plants for one experiment. **c** Final length of leaves 4, 5 and 6. Data are means ± sd of 90–106 plants (10–20 individuals in 6 experiments). * indicates significant effect of chilling (*P* < 0.05). **d** Leaf length deficit in chilling-treated plants, as % of controls

The F2 and F03802 contrasted Flint lines were chosen for further analyses in hybrid context. The F353 Dent line was chosen as the male tester, as it originates mainly from Iodent germplasm, a group well known for its good combining ability with Flint material [5]. Since we expected a higher number of leaves in hybrids as compared with the Flint parents and an heterotic effect on plant tolerance to low temperatures, chilling was applied at a later stage (6-VL) and in harder conditions (10 °C day/4 °C night) than in the inbred experiments. We observed that the rate of leaf emergence was similar in the hybrids than in the inbreds in control conditions, and that chilling caused a strong inhibition of growth (Fig. 2a). Plants of hybrid F03802xF353 completely stopped growing during the treatment whereas F2xF353 continued very slowly. Phyllochron rose to normal values upon return to warm conditions. Thus, the hybrids behaved as their Flint parental line F03802 and F2, respectively.

In the experiments reported above, the effect of cold was evaluated on the emergence of leaves, which occurs during their linear phase of elongation. This was confirmed by elongation rate measurements in the hybrid experiment performed at 6-VL stage (Fig. 2b). During cold treatment, elongation of leaf 6 immediately ceased in hybrid F03802xF353 but persisted at a very low rate in F2xF353, for about 4 days. In both hybrids, the elongation rate returned to its initial value upon return to warm temperature, but for a shorter time than the duration from appearance to the end of linear phase in control conditions. Consequently, the size of leaf 6 was significantly lower in cold-treated plants (Fig. 2c). The blade was relatively more affected than the sheath (Fig. 2d). The same measurements were performed on leaf 5, which was at the end of the linear phase of elongation at the start of the cold treatment, and on leaf 4 which had already entered the decelerating phase of leaf growth, i.e., had reached its final blade length. Cold caused a severe reduction of elongation in the two leaves but, again, F2xF353 kept on growing very slowly during the first days at low temperature whereas elongation completely stopped in F03802xF353. By contrast, recovery was better in the latter hybrid. Finally, leaf 5 was significantly shorter in cold-treated plants and the blade and sheath lengths were equally reduced by about 10 % (Fig. 2c and d). Only the sheath of leaf 4 was reduced after cold, but there was no consequential and significant impact on total leaf length. These effects of cold on leaf length were altogether insufficient to cause a significant reduction in shoot biomass measured at 10-VL (data not shown).

### Chilling effects on the photosynthetic machinery

We measured the maximum quantum efficiency of PSII photochemistry (Fv/Fm), which is a good indicator of PSII integrity [38] and the quantum yield of electron transport in the light (ΦPSII) at 300 (inbreds) or 400 (hybrids) μmol m^−2^ s^−1^ PAR. The use of excitation energy unaccounted for by ΦPSII was estimated as in [31] by ΦNPQ, which is the fraction of light that is dissipated thermally via regulated processes and by Φf,D, which is the fraction of light that is lost by fluorescence (Φf) or constitutive thermal dissipation (ΦD).

**Fig. 3:**
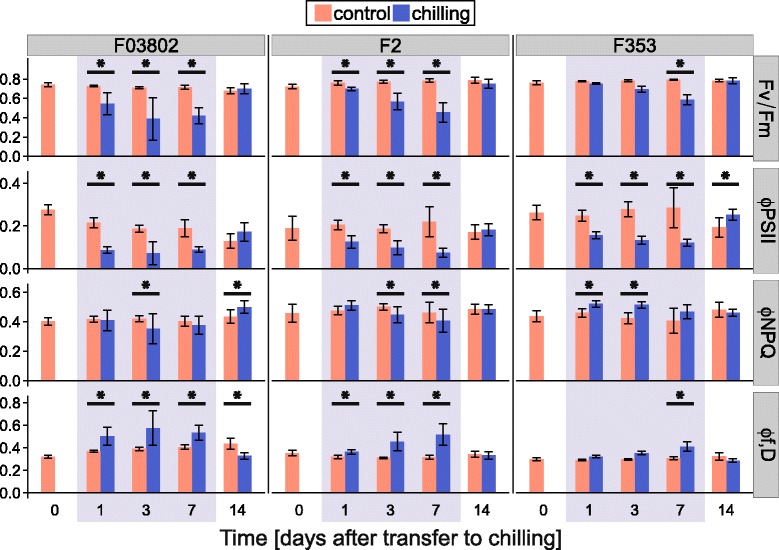
Effect of chilling on chlorophyll fluorescence parameters in 3 inbred lines of maize. The 7-day chilling treatment (10 °C day/7 °C night) was applied at about the 5-VL stage (grey zone). From the top to the bottom: maximal quantum yield of PSII (Fv/Fm); quantum yield of PSII in the light (300 μmol m^−2^ s^−1^ PAR, ΦPSII); regulated thermal energy dissipation (ΦNPQ); constitutive thermal and radiative (fluorescence) energy dissipation (Φf,D). Measurements were performed in the middle of 4th leaf blade before (day 0), during (days 1, 3 and 7) and after (day 14) the chilling treatment. Data are means ± sd of 5 plants for one experiment. * indicates significant effect of chilling (*P* < 0.05)

No significant difference was found between the three parental inbreds for Fv/Fm measurements in control conditions but more variability was found for ΦPSII, F2 being the least efficient inbred (Fig. 3). Chilling induced a severe reduction of Fv/Fm from the first day for the two Flint inbreds whereas F353 was significantly affected after one week of stress only. For the three inbreds, ΦPSII was negatively impacted by chilling from the onset, reflecting severe reduction of photochemical processes. Amazingly, dissipation of the excess light energy did not occur by regulated heat emission but by fluorescence or constitutive heat emission in the Flint inbreds F2 and F03802, as indicated by the decrease in ΦNPQ and the increase in Φf,D during chilling. The situation was different in the Dent inbred F353 where an increase in ΦNPQ, and hence in regulated thermal dissipation, was observed at the beginning of the chilling period and followed by an increase in Φf,D after 7 days of stress, when Fv/Fm was lower. It is then noteworthy that variations in Φf,D and Fv/Fm occurred in opposite patterns in all three inbreds. Seven days after the end of chilling (day 14, Fig. 3), all parameters had recovered to control levels for all inbreds, indicating that the damages caused by the treatment on the integrity and functioning of photosystems were reversible. For Dent inbred F353, ΦPSII of treated plant was even higher after chilling than that of control plants. This observation suggests a compensatory activation of photosynthesis after cold but might also be explained by a decline of photosynthesis efficiency in control conditions possibly due to faster leaf senescence.

**Fig. 4:**
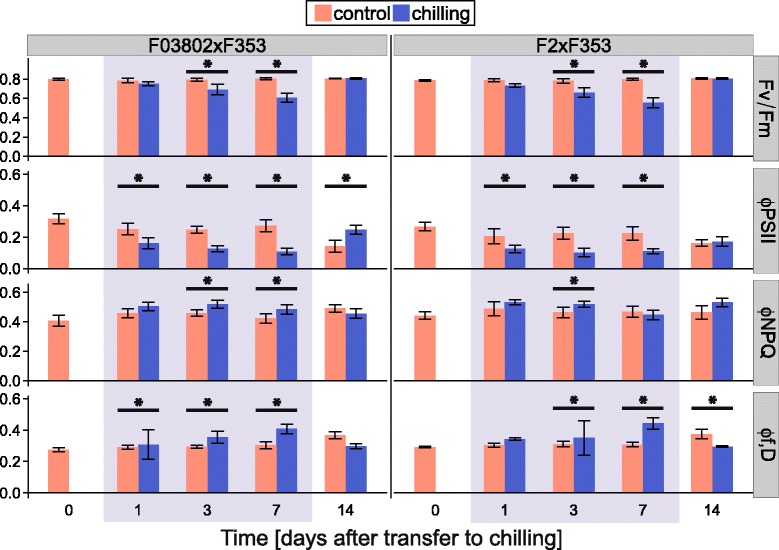
Effect of chilling on chlorophyll fluorescence parameters in two maize hybrids. The 7-day chilling treatment (10 °C day/4 °C night) was applied at about the 6-VL stage (grey zone). From top to bottom: maximal quantum yield of PSII (Fv/Fm); quantum yield of PSII in the light (400 μmol m^−2^ s^−1^ PAR, ΦPSII); regulated thermal energy dissipation (ΦNPQ); constitutive thermal and radiative (fluorescence) energy dissipation (Φf,D). Measurements were performed in the middle of 4th leaf blade before (day 0), during (days 1, 3 and 7) and after (day 14) the chilling treatment. Data are means ± sd of 29 plants (5–8 individuals in 4 experiments). * indicates significant effect of chilling (*P* < 0.05)

The same analysis was performed with the two hybrids F2xF353 and F03802xF353. As observed with the parents, low temperature had a negative effect on Fv/Fm and ΦPSII (Fig. 4). Interestingly though, the impact on Fv/Fm was significant after 3 days of chiling only, i.e., later than what was observed for the Flint parents F2 and F03802 (Fig. 3), suggesting a benefit from the F353 Dent parent. By contrast, ΦPSII had already decreased after 1 day of chilling, indicating a reduction in electron transport, and continued to do so until the end of the treatment. Most interestingly, dissipation of the excess energy occurred by regulated and non-regulated (constitutive) processes since both ΦNPQ and Φf,D increased during the chilling treatment. This result indicates that the hybrids combined the dissipation strategies of their Flint and Dent parents shown in Fig. 3.

Chlorophyll and carotenoid contents of leaf 4 were compared in treated and control plants (Table 2). Tissues were harvested at the end of the chilling period for the treated plants, or 1 day after the beginning of the treatment for the control plants to compare plants at the same VL stage. In control conditions, F03802xF353 contained more chlorophyll (*a* + *b*) per unit leaf area than F2xF353. Chilling caused a strong decrease in chlorophyll content in both hybrids, but the chlorophyll *a*/*b* ratio showed little variation. By contrast, there was a noticeable accumulation of the precursor geranylgeranyl chlorophyll *a*, indicating a reduction in chlorophyll *a* biosynthesis; this effect was stronger in F2xF353 than in F03802xF353. The carotenoid content of the leaf was not very different in chilling versus standard conditions but whereas the xanthophyll pool was almost fully epoxidized in control conditions, the de-epoxidation index (de-epoxidation of violaxanthin via antheraxanthin to zeaxanthin) increased to about 20 % in the cold, indicating the activation of the xanthophyll cycle. The index was slightly higher in F03802xF353 than in F2xF353.

**Table 2:** Effect of chilling treatment on leaf pigment content in two maize hybrids

	F03802xF353		F2xF353	
	Control	Chilling	Control	Chilling
chl *a* + *b* [μg.cm^−2^]	64 ± 14 b	49 ± 12 b	43 ± 8 a	32 ± 9 a
chl *a* / *b*	4.1 ± 0.1 b	4.3 ± 0.1 c	3.8 ± 0.2 a	4.1 ± 0.2 b
GG-chl *a* / chl *a* [‰]	0.8 ± 0.1 b	3.2 ± 0.4 c	0.2 ± 0.1 a	4.8 ± 0.3 d
total carotenoids / chl *a* + *b* [%]	10.6 ± 0.3 b	11.8 ± 1.6 b	9.1 ± 0.4 a	12.5 ± 1.8 ab
β carotene / chl *a* + *b* [%]	3.5 ± 0.2 c	3.2 ± 0.1 b	2.6 ± 0.2 a	2.8 ± 0.2 a
lutein / chl *a* + *b* [%]	4.0 ± 0.2 b	4.6 ± 0.7 b	3.3 ± 0.2 a	4.4 ± 0.8 ab
total xanthophylls / chl *a* + *b* [%]	2.2 ± 0.2 a	3.3 ± 0.9 a	2.3 ± 0.3 a	4.3 ± 0.9 b
de-epoxidation index [A+Z/V+A+Z, %]	1.3 ± 0.5 a	19.2 ± 4.9 c	3.3 ± 0.8 a	15.7 ± 4.4 b

*chl* chlorophyll, *GG* geranylgeranyl, *A* antheraxantin, *Z* zeaxanthin, *V* violaxanthin. The 7-day chilling treatment (10 °C day/4 °C night) was applied at about the 6-VL stage. Sampling was made on leaf 4 blade (15 cm from the ligule, on either side of the midrib) at the end of the chilling treatment for treated plants or 1 day after the beginning of the treatment for control plants in order to compare plants at the same developmental stage. Data are means ± sd of 21 plants (7 individuals in 3 experiments). Different letters indicate significant differences between groups (*P* < 0.05)

**Fig. 5:**
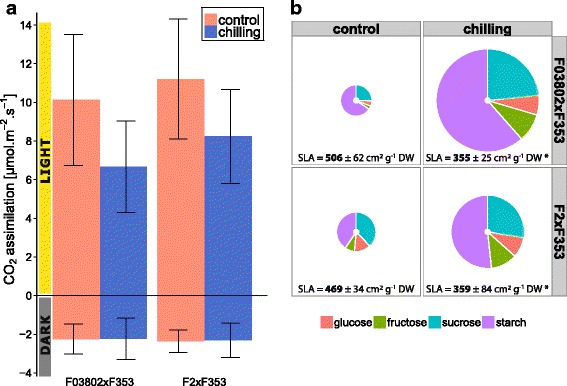
Effect of chilling on CO_2_ assimilation, leaf sugar content and SLA in two maize hybrids. The 7-day chilling treatment (10 °C day/4 °C night) was applied at about the 6-VL stage. Analyses were performed on the 4^th^ leaf blade at the end of the chilling treatment for treated plants or 1 day after the beginning of the treatment for control plants in order to compare plants at the same developmental stage. **a** CO_2_ assimilation measured in the light (400 μmol m^−2^ s^−1^ PAR) or in the dark at 25 °C, 380 μmol CO_2_ mol^−1^. Data are means ± sd of 15–20 plants (5–10 individuals in 2 experiments). **b** SLA, soluble sugars and starch quantification. Pie chart area is proportional to sugar amount (white disc in the center = 1 μg cm^−2^ scale). Different colours represent glucose (*pink*), fructose (*green*), sucrose (*blue*) and starch (*purple*). Data are means ± sd of 24 plants (8 individuals in 3 experiments). For SLA determination, data are means ± sd of 55–60 plants (15–20 individuals in 3 experiments)

With regard to the activity of the C4 cycle, CO_2_ assimilation of leaf 4 in the light was negatively impacted by cold treatment but respiration in the dark was not (Fig. 5a, Additional file 3: Figure S2). Starch and soluble sugars accumulated in the leaf of cold-treated plants (Fig. 5b). This was particularly marked in F03802xF353 hybrid that accumulated about 10 times more starch, sucrose and glucose in chilling conditions than in controls whereas the amplitude of variation was between 2 and 4 in F2xF353. In both hybrids, SLA was lower after cold than in control conditions (Fig. 5b), which is consistent with the accumulation of carbohydrates.

### Effects of low temperature on gene expression

**Fig. 6:**
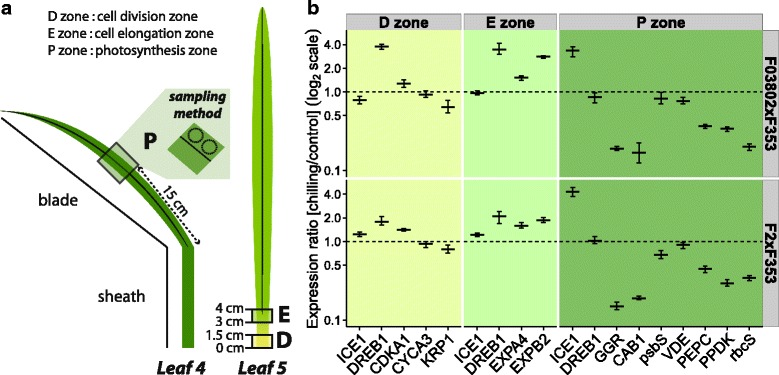
Effect of chilling on gene expression in the leaves of two maize hybrids. The 7-day chilling treatment (10 °C day/4 °C night) was applied at about the 6-VL stage. Analyses were performed at the end of the chilling treatment for treated plants or 1 day after the beginning of the treatment for control plants in order to compare plants at the same developmental stage. **a** Sampling procedure. **b** Relative expression levels quantified by RT-qPCR. Five genes were used for normalization. Data are means ± se of 3 technical replicates for one experiment; two replicate experiments are shown in Additional file 4: Figure S3. Gene abbreviations: *ICE1* (*INDUCER OF CBF/DREB EXPRESSION 1*), *DREB1* (*DROUGHT*-*RESPONSIVE ELEMENT BINDING*), *CDKA1* (*CYCLIN DEPENDENT KINASE A 1*), *CYCA3* (*CYCLIN A 3*), *KRP1* (*CYCLIN-DEPENDENT KINASE INHIBITOR 1*), *EXPA4* (*ALPHA EXPANSIN 4*), *EXPB2* (*BETA EXPANSIN 2*), *GGR* (*GERANYLGERANYL REDUCTASE*), *CAB1* (*CHLOROPHYLL A/B BINDING PROTEIN*), *psbS* (CP22 PSII subunit), *VDE* (*VIOLAXANTHIN DE-EPOXIDASE*), *PEPC* (*PHOSPHOENOLPYRUVATE CARBOXYLASE*), *PPDK* (*PYRUVATE*, *ORTHOPHOSPHATE DIKINASE*) and *rbcS* (*RUBISCO* small subunit)

In order to see whether the effects of cold on leaf elongation and photosynthesis could be correlated with changes in gene expression, a panel of candidate genes was selected from the literature and analysed by RT-qPCR. Two samples were taken in the growing zone of leaf 5: one in the meristematic zone and one in the elongation zone (Fig. 6a). For photosynthetic metabolism, leaf discs were harvested in the blade of leaf 4 where the Fv/Fm, ΦPSII, CO_2_ assimilation, pigments and sugar analyses had been performed. In all zones, the transcript levels of *DREB1* and *ICE1* homologs were quantified as markers of cold signalling [16, 39] and five different constitutive genes were used for relative RT-qPCR quantifications. We found that *ZmDREB1* was induced at the end of the chilling treatment in the cell division and cell elongation zones of leaf 5, whereas the transcript levels of *ICE1* were similar than in untreated leaves (Fig. 6, Additional file 4: Figure S3). The up-regulation of *ZmDREB1* was two times higher in F03802xF353 than in F2xF353. Interestingly, the opposite was observed in the mature zone of leaf 4, where *ICE1* was strongly induced in low temperature conditions but not *ZmDREB1*. These results suggest that chilling signalling is different in growing non-photosynthetic tissues and in photosynthetic cells.

The transcripts of three cell-cycle genes were quantified in the proliferation zone of leaf 5: *CDKA1* (*CYCLIN DEPENDENT KINASE A 1*), *CYCA3* (*CYCLIN A 3*) and *KRP1* (*CYCLIN-DEPENDENT KINASE INHIBITOR 1*) were chosen after Rymen et al. [15] showed that these genes were differentially expressed in the meristematic zone of leaves exposed to cold nights. This was obviously not the case in our experimental design since the relative transcript levels of the three genes were hardly different in chilled and control leaves. In the elongation zone, two *EXPANSIN* genes, *EXPA4* and *EXPB2*, selected from [40] were up-regulated in the cold. In the mature zone of leaf 4, the list of candidates genes included *CAB1*, encoding a CHLOROPHYLL A/B BINDING PROTEIN of PSII [22], *psbS* and *VIOLAXANTHIN DE-EPOXIDASE* (*VDE*) both involved in energy dissipation (ΦNPQ) (reviewed in [27]), the gene encoding *GERANYLGERANYL REDUCTASE* (*GGR*) that catalyses the terminal hydrogenation of geranylgeranyl chlorophyll *a* to form chlorophyll *a* [41] and genes encoding C4- and Calvin-cycle enzymes: PEPC, PPDK and the small subunit of RUBISCO (rbcS). Results were fully consistent for both hybrids: we observed a strong downregulation of *CAB1* and *GGR* in the chilling conditions, but no change in the relative transcript level of *psbS* and *VDE*. The genes encoding enzymes of the C4 metabolism were all downregulated in the cold.

## Discussion

The aim of this study was to analyse the mechanisms of cold tolerance in European maize in order to identify appropriate ways towards further improvement. Although the genetic panel was small, two levels of response to chilling were observed (Fig. 1): either a complete stop in vegetative growth during the chilling treatment (1 week at 10 °C day/7 °C night) as observed in one Flint inbred (F03802) and two Dents (B73 and Mo17), or a very slow continuation of growth, recorded in two Flint lines (F2 and F283) and one Dent (F353). Cessation of growth was consistent with the fact that 10 °C is basal temperature for maize [42]. Interestingly, the habit of the Flint parent was transmitted in the Flint x Dent hybrid, as observed after the F03802xF353 and F2xF353 crosses (Fig. 2a). The analyses were thereafter continued on the hybrids.

Cessation of growth was measured as of lag in leaf appearance and hence could be due to cessation of cell proliferation or expansion in the emerging leaf. Rymen et al. [15] observed that cold nights (25 °C day/4 °C night cycles) reduced maize leaf growth through a prolonged cell cycle in the meristematic zone and correlated this with changes in the expression level of cell cycle genes. Using three of their markers, we did not observe any change in transcript level in our experimental conditions (Fig. 6, Additional file 4: Figure S3), which differed from Rymen’s in the temperature regime and the developmental stage of the leaf. Louarn et al. [21] indeed found that chilling impairs cell proliferation during the exponential phase of leaf growth (as in [15]) and cell elongation during the late linear phase of growth. The reduction of cell elongation is likely to be responsible for the 8-fold reduction in elongation rate observed in our study. It is then all the more surprising that we detected an increased level of transcripts for two expansin genes in the leaves that were exposed to low temperatures. While expansins have primarily a growth promoting function, they seem important for counteracting growth-repressing activities [43, 44]. Moreover, up-regulation of expansin genes was described in Arabidopsis leaves exposed to a mild drought stress, opposite to their down-regulation in severe drought, and was interpreted as a ‘get ready for growth’ state [45]. Such a hypothesis would perfectly fit chilling tolerance in the maize hybrids since we observed a very fast resumption of growth upon return of the plants to warm temperatures (Fig. 2).

A reduction in temperature slows down most physiological processes, explaining the arrest of growth. Decreased sink activity in turn can alter carbohydrate allocation and if assimilation of CO_2_ continued in the photosynthetic tissues, carbohydrates would accumulate. This scenario was observed in both hybrids, in which chilling caused a reduction in CO_2_ assimilation but a sharp increase in sugar content and consequently a decrease in SLA (Fig. 5, Additional file 3: Figure S2). Interestingly, these changes were of higher amplitude in the F03802xF353 hybrid, which completely ceased growing during chilling, than in F2xF353, indicating a correlation with growth rate. Similar phenotypic differentiation was found between European Flint and European Dent breeding groups by GWAS [29]. In this study, the authors reported that the specific response of European Flint lines to chilling temperatures was a strong reduction in SLA, i.e., a rather xeromorphic growth habit resulting from the reduction in leaf area with only little reduction in leaf dry weight, and suggested that this might be a way to reduce transpiration. By contrast, the specific adaptation of European Dent to chilling stress appeared to be the maintenance of growth despite reduced photosynthetic capacity, suggesting a highly efficient carbon fixation. These two strategies appeared in our study as differentiating the F03802xF353 and F2xF353 hybrids, although their distinct parents were from the same European Flint breeding group. A possibility could be that the Dent traits were differentially expressed in the two hybrids but the phenotypic differentiation in terms of growth cessation or continuation was observed in the parental F03802 and F2 lines, indicating differentiation within the European Flint breeding group.

The decrease in CO_2_ assimilation in maize plants grown at low temperatures is abundantly documented [46]. It is at least partly a primary effect of low temperatures limiting chemical reaction rates catalysed by C4- and Calvin-cycle enzymes [47, 48]. However, we detected a reduction in the relative abundance of transcripts encoding three major proteins: PEPC, PPDK and rbcS (Fig. 6, Additional file 4: Figure S3), indicating that photosynthetic genes were downstream targets of cold signalling, either direct or indirect by negative feedback from accumulating sugars (reviewed in [49]). Surprisingly, cold signalling in the mature leaf of the two hybrids where these changes were analysed did not follow the canonical pathway described in Arabidopsis where *ICE1* is constitutively expressed and activation of the ICE1 protein by cold is responsible for up-regulation of *DREB1* gene [50]. On the opposite, we observed up-regulation of *ICE1* but not of *ZmDREB1* in the mature leaf exposed to chilling whereas the reverse pattern was observed in proliferation and elongation zones of younger leaf (Fig. 6, Additional file 4: Figure S3). Hu et al. [16] showed that cold triggered chromatin modifications at the ICE1 binding region of *ZmDREB1* promoter in maize seedlings, where both *ZmDREB1* and *ICE1* are strongly induced. Further experiments are required to understand the developmental changes in cold-signalling pathway in maize leaves.

The rate of consumption of NADPH and ATP are major factors that determine PSII operating efficiency and hence the decrease in CO_2_ assimilation could account for the decreased PSII operating efficiency (ΦPSII) detected in chilling conditions. We indeed observed a reduction in ΦPSII before any symptom of photoinhibition, such as a decrease in Fv/Fm (Fig. 4). When the rate of photosynthesis is reduced by chilling, excess excitation energy has to be dissipated either by constitutive or light-activated processes (Φf,D and ΦNPQ) [31, 51]. Surprisingly, we observed that the Flint inbreds F2 and F03802 mostly dissipated the energy by constitutive processes (heat and fluorescence) whereas the Dent F353 activated heat dissipative NPQ processes. Even more interesting, the Flint x Dent hybrids appeared to use both parental strategies, but to moderate levels. We did not observe a strong enhancement of ΦNPQ during chilling treatment of the two hybrids (Fig. 4); the size of the xanthophyll pool did not increase and even if the de-epoxidation index rose to about 20 % (Table 2), this level remained much lower than what can be found in the literature (70-80 % in [22, 52]). We did not detect any effect of chilling on the relative abundance of transcripts encoding the psbS or VDE proteins that are involved in regulated heat dissipation (Fig. 6, Additional file 4: Figure S3). Although we can not dismiss post-translational regulation of these proteins [53], the weak variations in ΦNPQ and Φf,D suggest that the photosynthetic machinery of the hybrids was not much impacted by the chilling treatment.

Severe chilling stress leads to destruction of the PSII reaction center protein D1 and a decrease in chlorophyll *a*/*b* ratio was seen as a sign of the progressive imbalance between antennas and reaction center integrity [22, 54]. Again, we did not observe this symptom and hence the decrease in Fv/Fm that we measured would be an indicator of PSII inactivation rather than photoinhibition *per se*. Interestingly, we observed that chilling compromised expression of *CAB1* gene (Fig. 6, Additional file 4: Figure S3), encoding one of the major proteins of PSII antennas, as well as chlorophyll biosynthesis (Table 2). These can be seen as adaptation mechanisms to reduce antennas size and light absorption.

## Conclusions

In conclusion our study suggests that the photosynthetic machinery of European maize is pretty well adapted to short chilling spells and hence that further improvements may not originate from this avenue. By contrast, the growth habit might have been underestimated so far since, from a breeding point of view, strategies to achieve greater biomass at low temperatures require sustained growth at low temperature [55]. This rationale might be reversed if refraining growth during chilling maintained the plants in a ‘ready to grow’ state to ensure fast recovery once better temperatures are restored. New adaptation traits could then be selected for areas with short cold events by considering the ability of the mechanisms regulating cell proliferation and cell expansion in the growing zones of the leaves to maintain a standby mode during the stress.

## Additional files

Additional file 1: Table S1. List of primers used in RT-qPCR experiments. All genes chosen in the literature were identified in the NCBI website (http://www.ncbi.nlm.nih.gov/) and specific primers were designed using PrimerBLAST (http://www.ncbi.nlm.nih.gov/tools/primer-blast/). Primer pairs were validated for secondary structures at 60 and 72 °C using the MFOLD web server (http://unafold.rna.albany.edu/?q=mfold/DNA-Folding-Form) and tested for gene specificity in PCR and amplification efficiency in qPCR on successively diluted template cDNA. (PDF 8 kb)
Additional file 2: Figure S1. Identification of constitutive genes for RT-qPCR analyses. Ten housekeeping genes (see Additional file 1: Table S1) were tested for constitutive expression in a mix of leaf tissues taken along the developmental gradient of leaf 5 (base, middle and tip) and at the tip of leaf 6 in control and chilled plants. 8–15 plants were pooled for RT-qPCR. A geNormPLUS analysis was performed for the calculation of the M value for each gene. The dotted line indicates the threshold below which the gene is regarded as ‘constitutive’ in this set of samples (geNorm M value < 0.5). Gene abbreviations: *ACT1* (*ACTIN 1*), *GPA1* (*GLYCERALDEHYDE-3-PHOSPHATE DEHYDROGENASE*), *TUB* (*TUBULIN ALPHA 3 CHAIN*), *EF1A* (*ELONGATION FACTOR 1 ALPHA*), *MEP* (*MEMBRANE PROTEIN*), *FGP1* (*FOLYLPOLYGLUTAMATE SYNTHASE*), *UBI* (*UBIQUITIN*), *LUG* (*LEUNIG*), *CUL* (*CULLIN*) and *UCE* (*UBIQUITIN CONJUGATING ENZYME* E2). (PDF 311 kb)
Additional file 3: Figure S2. Effect of chilling on CO_2_ assimilation in two maize hybrids. The 7-day chilling treatment (10 °C day/4 °C night) was applied at about the 6-VL stage. Measurements were performed in the middle of 4th leaf blade at the end of the chilling treatment for treated plants or 1 day after the beginning of the treatment for control plants in order to compare plants at the same developmental stage. Ambient parameters were 25 °C, 380 μmol CO_2_ mol^−1^. (a) Irradiance response curves of CO_2_ assimilation. The irradiances used for the measurements were 2000, 1500, 1000, 800, 600, 400, 300, 150 and 0 μmol m^−2^ s^−1^ PAR. (b) Curve parameters. Initial slope was calculated between irradiance 0 and 150 μmol m^−2^ s^−1^ PAR; compensation point is the irradiance at which CO_2_ assimilation is 0. Data are means ± sd of 15–20 plants (5–10 individuals in 2 experiments). (PDF 349 kb)
Additional file 4: Figure S3. Effect of chilling on gene expression in the leaves of two maize hybrids. The 7-day chilling treatment (10 °C day/4 °C night) was applied at about the 6-VL stage. Analyses were performed at the end of the chilling treatment for treated plants or 1 day after the beginning of the treatment for control plants in order to compare plants at the same developmental stage. **a** and **b** are biological replicates of Fig. 6. Data are means ± se of 3 technical replicates. Gene abbreviations: *ICE1* (*INDUCER OF CBF/DREB EXPRESSION 1*), *DREB1* (*DROUGHT-RESPONSIVE ELEMENT BINDING*), *CDKA1* (*CYCLIN DEPENDENT KINASE A 1*), *CYCA3* (*CYCLIN A 3*), *KRP1* (*CYCLIN-DEPENDENT KINASE INHIBITOR 1*), *EXPA4* (*ALPHA EXPANSIN 4*), *EXPB2* (*BETA EXPANSIN 2*), *GGR* (*GERANYLGERANYL REDUCTASE*), *CAB1* (*CHLOROPHYLL A/B BINDING PROTEIN*), *psbS* (CP22 PSII subunit), *VDE* (*VIOLAXANTHIN DE-EPOXIDASE*), *PEPC* (*PHOSPHOENOLPYRUVATE CARBOXYLASE*), *PPDK* (*PYRUVATE, ORTHOPHOSPHATE DIKINASE*) and *rbcS* (*RUBISCO* small subunit). (PDF 351 kb)

## Abbreviations

*A*: Antheraxanthin
*CAB1*: Chlorophyll a/b binding protein 1
*CDKA1*: Cyclin dependent kinase A 1
*chl*: Chlorophyll
*CYCA3*: Cyclin A 3
*DREB1*: Drought-responsive element binding 1
*EXPA4*: Alpha expansin 4
*EXPB2*: Beta expansin 2
*GG*: Geranyl geranyl
*GGR*: Geranyl geranyl reductase
*ICE1*: Inducer of CBF/DREB expression 1
*KRP1*: Cyclin-dependent kinase inhibitor 1
*PEPC*: Phosphoenolpyruvate carboxylase
*PPDK*: Pyruvate orthophosphate dikinase
*psbS*: Chloroplast protein 22 PSII subunit
*PSII*: Photosystem II
*rbcS*: Ribulose-1,5-bisphosphate carboxylase/oxygenase small subunit
*RT-qPCR*: Reverse transcription real-time quantitative PCR
*SLA*: Specific leaf area
*V*: Violaxanthin
*VDE*: Violaxanthin deepoxidase
*VL*: Visible leaf stage
*Z*: Zeaxanthin
