# Supplementary material for: Maize plants can enter a standby mode to cope with chilling stress
Source: BMC Plant Biol. 2016 Oct 4;16:212. doi: 10.1186/s12870-016-0909-y (PMC5050578; doi:10.1186/s12870-016-0909-y)
Supplement: Additional file 1: Table S1. — List of primers used in RT-qPCR experiments. All genes chosen in the literature were identified in the NCBI website (http://www.ncbi.nlm.nih.gov/) and specific primers were designed using PrimerBLAST (http://www.ncbi.nlm.nih.gov/tools/primer-blast/). Primer pairs were validated for secondary structures at 60 and 72 °C using the MFOLD web server (http://unafold.rna.albany.edu/?q=mfold/DNA-Folding-Form) and tested for gene specificity in PCR and amplification efficiency in qPCR on successively diluted template cDNA. (PDF 8 kb) [file 12870_2016_909_MOESM1_ESM.pdf]

Additional file 1: Table S1

Name	Type of gene	Amplification efficiency [%]	Tm [°C]	Forward primer sequence	Reverse primer sequence	Reference
ACT1	Constitutive	105	57	GGTGCTCCCTGCTGTATGAA	AGGCAACACGTTACACCAGT	Sekhon *et al*. (2011)
GPA1	Constitutive	82	58	GGCGTTAATGCCGACCAGTA	TAGGAGTGGGTGGTGGTCAT	Sekhon *et al*. (2011)
TUB	Constitutive	80	57	ATCTATGACATCTGCCGCCG	GGAGCGTAGGACGAAAGCAT	Sekhon *et al*. (2011)
EF1A	Constitutive	82	57	AAGTATGCGTGGGTGCTTGA	GGTCTCGAACTTCCACAGGG	Sekhon *et al*. (2011)
MEP	Constitutive	106	57	GCCAAGATCTTCTGCTGGGT	GAACGAGAAGGAACAGCCCA	Manoli *et al*. (2012)
FGP1	Constitutive	97	58	AGTGATACGCCGCTCGAAAT	GGCACCAATAAGCAAGCACC	Manoli *et al*. (2012)
UBI	Constitutive	85	58	CCCATGGATGGTGCTGTCTT	GCGGTCGCACGATAGTTTTG	Sekhon *et al*. (2011)
LUG	Constitutive	107	58	AGTGCTACAGGGAAGGTTGC	TCATGTGCGTCATGTGGTCA	Manoli *et al*. (2012)
CUL	Constitutive	92	57	GCAGGAAGATTGCCATCCCA	ACCTTGCCTGATTGGTGGTT	Manoli *et al*. (2012)
UCE	Constitutive	104	57	CCGACAGGGCCAAGTATGAG	CCGGCACATTCACCAGTTTG	Sekhon *et al*. (2011)
ICE1	Candidate	91	57	CAACCCATCAACACCGAC	GCAAAGCCATTGAAGCAG	Hu *et al*. (2011)
DREB1	Candidate	117	57	CCAGCGGTAGTTGTTGAC	TGTTCCCGTTACATTCGT	Hu *et al*. (2011)
CDKA1	Candidate	107	57	CTGCCCGGAGTTTGCTAAGA	CGCAACACCGTGGAGTATCT	Rymen *et al*. (2007)
CYCA3	Candidate	103	57	ACCAGGCATTCTCTTGCTGT	CACGAGTAAAGTATCCACGCA	Rymen *et al*. (2007)
KRP1	Candidate	96	57	CGTCGTACGGGGAGAACAT	GTGGACCCAGGAGTGCTAGT	Rymen *et al*. (2007)
EXPA4	Candidate	101	57	TGTGTTAGAGGGTGGTGGGT	AGGACCTGGACCTGGGATTA	Muller *et al*. (2007)
EXPB2	Candidate	97	57	ATACTACGGCGGAACGGAAC	CCAAGAGACGCCTCAACCTT	Muller *et al*. (2007)
GGR	Candidate	111	58	ATCGACTGGATTCTGACGCC	AACCAGAACGGGGGAGTTTC	Tanaka *et al*. (1999)
CAB1	Candidate	91	57	AGAACCTCGCTGACCACATC	AGACACACATGCATCTCGCA	Caffarri *et al*. (2005)
psbS	Candidate	104	57	GGGATCGGTTTCACCAAGGA	CCGGTAATGGCCTCTCCAAG	Reviewed in Baker (2008)
VDE	Candidate	102	57	AGTGGCTTAAACCCGACGTT	TGCGGACTCTCCATGTGATG	Reviewed in Baker (2008)

Name	Type of gene	Amplification efficiency [%]	Tm [°C]	Forward primer sequence	Reverse primer sequence	Reference
PEPC	Candidate	98	57	CGAAGAGCTCCACAGTTCGT	GCTTGTCCCTTACATGGCCT	Naidu *et al*. (2003)
PPDK	Candidate	108	57	CTTTCATGGCCTGGGTGGAT	CCTTGTGCCCCATTGTTTCG	Naidu *et al*. (2003)
rbcS	Candidate	105	57	AGTAGCTAGTGCCATGCGTC	CAAGCAAGCAAAGGGTACGG	Naidu *et al*. (2003)
