# Supplementary figures and images for: Maize plants can enter a standby mode to cope with chilling stress
Source: BMC Plant Biol. 2016 Oct 4;16:212. doi: 10.1186/s12870-016-0909-y (PMC5050578; doi:10.1186/s12870-016-0909-y)

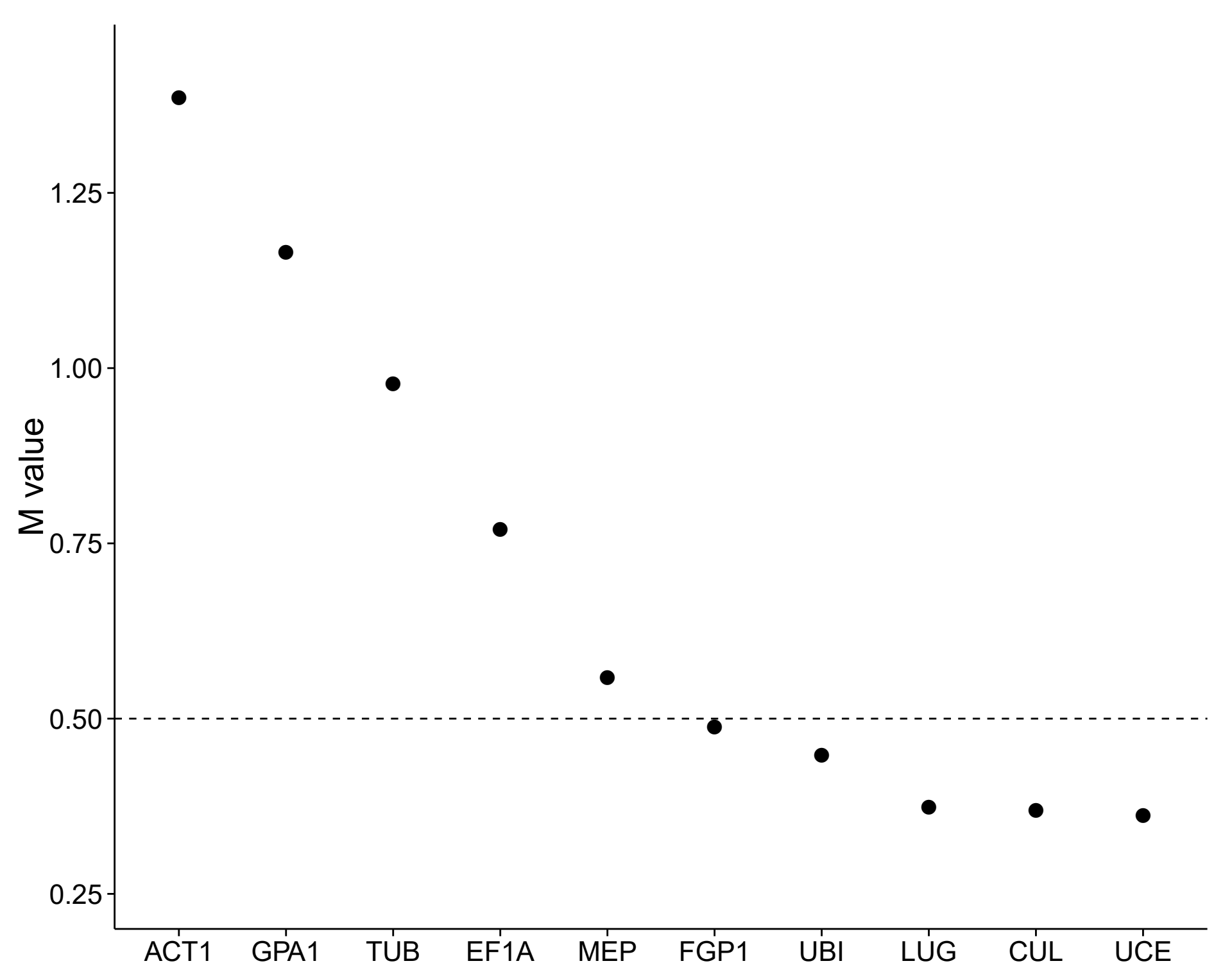

Supplement: Additional file 2: Figure S1. — Identification of constitutive genes for RT-qPCR analyses. Ten housekeeping genes (see Additional file 1: Table S1) were tested for constitutive expression in a mix of leaf tissues taken along the developmental gradient of leaf 5 (base, middle and tip) and at the tip of leaf 6 in control and chilled plants. 8–15 plants were pooled for RT-qPCR. A geNormPLUS analysis was performed for the calculation of the M value for each gene. The dotted line indicates the threshold below which the gene is regarded as ‘constitutive’ in this set of samples (geNorm M value < 0.5). Gene abbreviations: ACT1 (ACTIN 1), GPA1 (GLYCERALDEHYDE-3-PHOSPHATE DEHYDROGENASE), TUB (TUBULIN ALPHA 3 CHAIN), EF1A (ELONGATION FACTOR 1 ALPHA), MEP (MEMBRANE PROTEIN), FGP1 (FOLYLPOLYGLUTAMATE SYNTHASE), UBI (UBIQUITIN), LUG (LEUNIG), CUL (CULLIN) and UCE (UBIQUITIN CONJUGATING ENZYME E2). (PDF 311 kb) [file 12870_2016_909_MOESM2_ESM.pdf]

**a**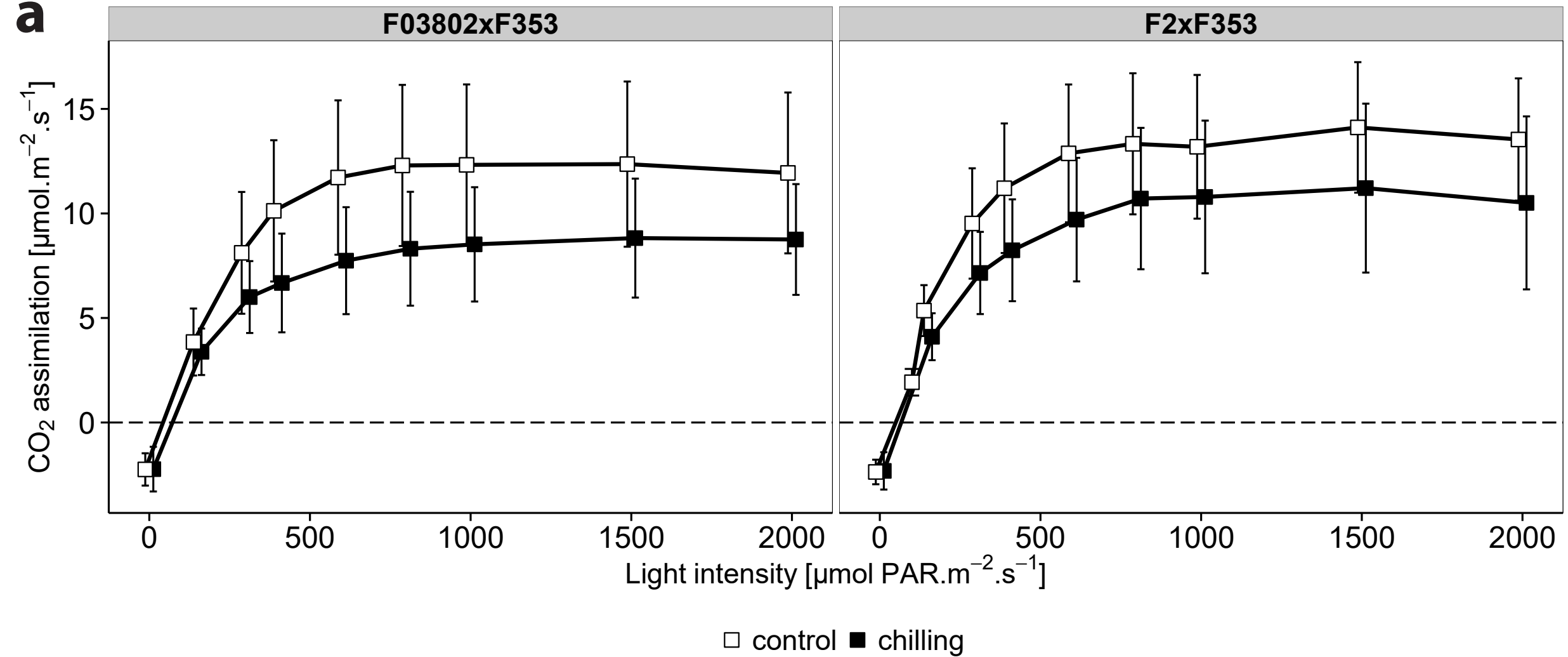**b**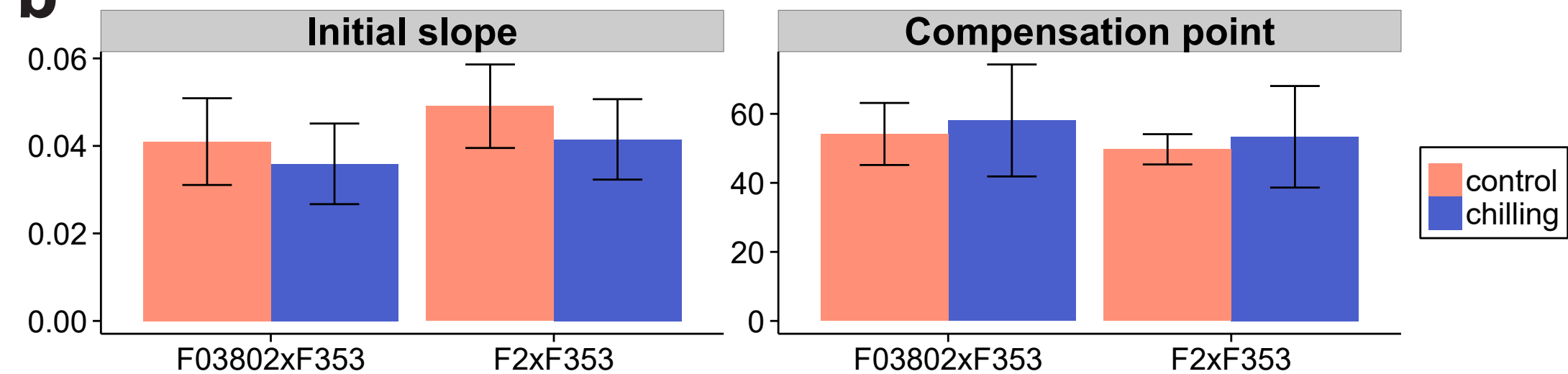

Supplement: Additional file 3: Figure S2. — Effect of chilling on CO2 assimilation in two maize hybrids. The 7-day chilling treatment (10 °C day/4 °C night) was applied at about the 6-VL stage. Measurements were performed in the middle of 4th leaf blade at the end of the chilling treatment for treated plants or 1 day after the beginning of the treatment for control plants in order to compare plants at the same developmental stage. Ambient parameters were 25 °C, 380 μmol CO2 mol−1. (a) Irradiance response curves of CO2 assimilation. The irradiances used for the measurements were 2000, 1500, 1000, 800, 600, 400, 300, 150 and 0 μmol m−2 s−1 PAR. (b) Curve parameters. Initial slope was calculated between irradiance 0 and 150 μmol m−2 s−1 PAR; compensation point is the irradiance at which CO2 assimilation is 0. Data are means ± sd of 15–20 plants (5–10 individuals in 2 experiments). (PDF 349 kb) [file 12870_2016_909_MOESM3_ESM.pdf]

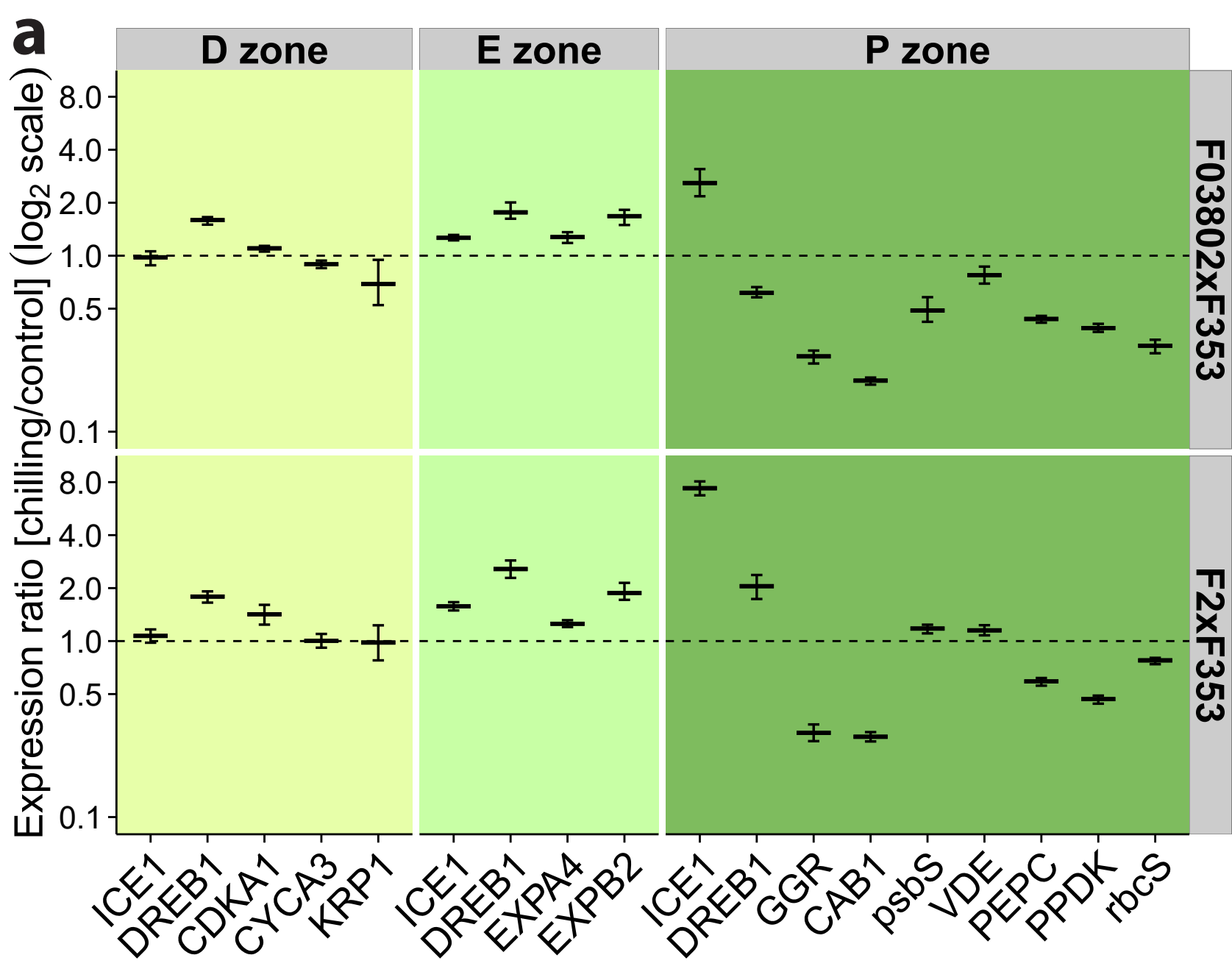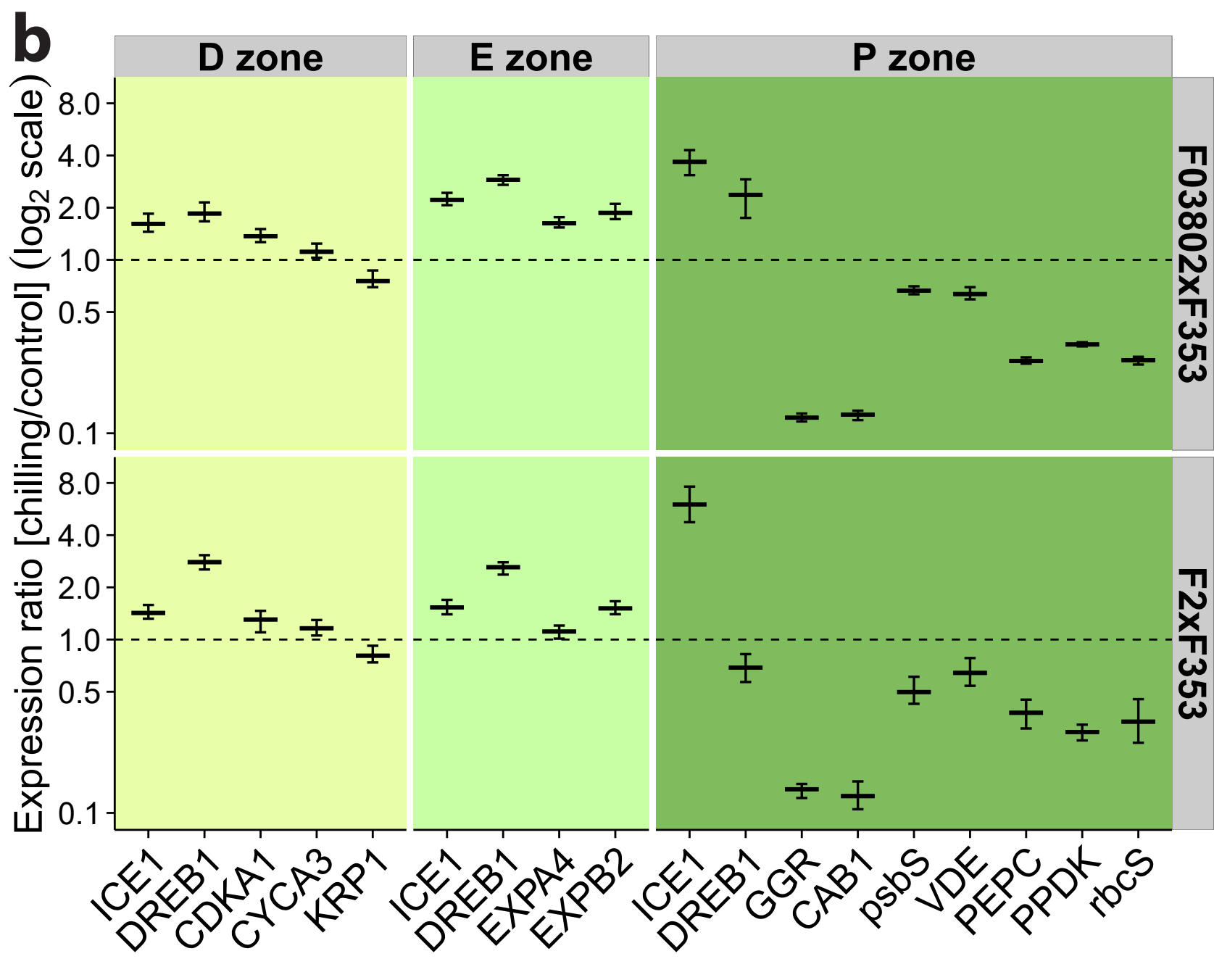

Supplement: Additional file 4: Figure S3. — Effect of chilling on gene expression in the leaves of two maize hybrids. The 7-day chilling treatment (10 °C day/4 °C night) was applied at about the 6-VL stage. Analyses were performed at the end of the chilling treatment for treated plants or 1 day after the beginning of the treatment for control plants in order to compare plants at the same developmental stage. a and b are biological replicates of Fig. 6. Data are means ± se of 3 technical replicates. Gene abbreviations: ICE1 (INDUCER OF CBF/DREB EXPRESSION 1), DREB1 (DROUGHT-RESPONSIVE ELEMENT BINDING), CDKA1 (CYCLIN DEPENDENT KINASE A 1), CYCA3 (CYCLIN A 3), KRP1 (CYCLIN-DEPENDENT KINASE INHIBITOR 1), EXPA4 (ALPHA EXPANSIN 4), EXPB2 (BETA EXPANSIN 2), GGR (GERANYLGERANYL REDUCTASE), CAB1 (CHLOROPHYLL A/B BINDING PROTEIN), psbS (CP22 PSII subunit), VDE (VIOLAXANTHIN DE-EPOXIDASE), PEPC (PHOSPHOENOLPYRUVATE CARBOXYLASE), PPDK (PYRUVATE, ORTHOPHOSPHATE DIKINASE) and rbcS (RUBISCO small subunit). (PDF 351 kb) [file 12870_2016_909_MOESM4_ESM.pdf]
